# Epidemiology and microbiology of ventilator-associated pneumonia in COVID-19 patients: a multicenter retrospective study in 188 patients in an un-inundated French region

**DOI:** 10.1186/s13054-021-03493-w

**Published:** 2021-02-18

**Authors:** Gauthier Blonz, Achille Kouatchet, Nicolas Chudeau, Emmanuel Pontis, Julien Lorber, Anthony Lemeur, Lucie Planche, Jean-Baptiste Lascarrou, Gwenhael Colin

**Affiliations:** 1 Médecine Intensive Réanimation, Centre Hospitalier Départemental de Vendee, Les Oudairies, 85000 La Roche-Sur-Yon, France; 2 Medical Intensive Care Unit, University Hospital of Angers, 4 rue Larrey 49933, Angers, France; 3 Médecine Intensive Réanimation, Centre Hospitalier Le Mans, 194 Avenue Rubillard, 72037 Le Mans, France; 4 Médecine Intensive Réanimation, Centre Hospitalier de Laval, 33, rue du Haut Roche, Laval, 53015 Canada; 5 Médecine Intensive Réanimation, Centre Hospitalier de Saint-Nazaire, 11 Boulevard Georges Charpak, 44600 Saint-Nazaire, France; 6 Médecine Intensive Réanimation, Centre Hospitalier de Cholet, 1 Rue de Marengo, 49300 Cholet, France; 7 Clinical Research Unit, Centre Hospitalier Départmental de Vendée, Les Oudairies, 85000 La Roche-Sur-Yon, France; 8 Médecine Intensive Réanimation, University Hospital of Nantes, 1 Place Alexis-Ricordeau, 44000 Nantes, France

**Keywords:** COVID-19, SARS-CoV-2, Acute respiratory distress syndrome, Ventilator-associated pneumonia

## Abstract

**Background:**

The COVID-19 pandemic is responsible for many hospitalizations in intensive care units (ICU), with widespread use of invasive mechanical ventilation (IMV) which exposes patients to the risk of ventilator-associated pneumonia (VAP). The characteristics of VAP in COVID-19 patients remain unclear.

**Methods:**

We retrospectively collected data on all patients hospitalized for COVID-19 during the first phase of the epidemic in one of the seven ICUs of the Pays-de-Loire region (North-West France) and who were on invasive mechanical ventilation for more than 48 h. We studied the characteristics of VAP in these patients. VAP was diagnosed based on official recommendations, and we included only cases of VAP that were confirmed by a quantitative microbiological culture.

**Findings:**

We analyzed data from 188 patients. Of these patients, 48.9% had VAP and 19.7% experienced multiple episodes. Our study showed an incidence of 39.0 VAP per 1000 days of IMV (until the first VAP episode) and an incidence of 33.7 VAP per 1000 days of IMV (including all 141 episodes of VAP). Multi-microbial VAP accounted for 39.0% of all VAP, and 205 pathogens were identified. Enterobacteria accounted for 49.8% of all the isolated pathogens. Bacteremia was associated in 15 (10.6%) cases of VAP. Pneumonia was complicated by thoracic empyema in five cases (3.5%) and by pulmonary abscess in two cases (1.4%). Males were associated with a higher risk of VAP (sHR 2.24 CI95% [1.18; 4.26] *p* = 0.013).

**Interpretation:**

Our study showed an unusually high incidence of VAP in patients admitted to the ICU for severe COVID-19, even though our services were not inundated during the first wave of the epidemic. We also noted a significant proportion of enterobacteria. VAP-associated complications (abscess, empyema) were not exceptional.

**Registration:**

As an observational study, this study has not been registered.

## Background

The COVID-19 pandemic caused by the SARS-CoV-2 virus is currently affecting many countries worldwide. Approximately 5% of patients with respiratory impairment develop a severe form with acute respiratory failure and require specialized management in the intensive care unit (ICU). The disease preferentially affects men over the age of 50 with cardiovascular risk factors (overweight, high blood pressure, diabetes), heart failure and/or respiratory comorbidities (chronic bronchitis, asthma) [1]. The characteristics are comparable for the majority of patients, who develop an acute respiratory distress syndrome (ARDS) with ground-glass X-ray opacities and a very intense systemic inflammatory response syndrome [2]. Since a few weeks after the beginning of March 2020, several thousand people have been receiving invasive mechanical ventilation (IMV) in France due to severe COVID-19.

IMV exposes patients to a particular risk of a nosocomial infectious complication called ventilator-associated pneumonia (VAP) [3]. In Europe, the incidence density is 18.3 VAP per 1000 days of IMV [4].

A French multicenter prospective epidemiologic ancillary study (from the ACURASYS [5] randomized trial) was conducted in 339 severe ARDS patients ventilated according to a lung-protective strategy between 2006 and 2008. In this study, 28.9% of the patients had at least one episode of microbiologically established bacterial VAP [6]. The mortality attributable to VAP is still being debated, but recent studies estimated a range of 4.4–9% [7, 8].

Informal exchanges between clinicians regarding the current pandemic indicate a high frequency of VAP. Several factors may account for a higher incidence of VAP in the population hospitalized in the ICU for SARS CoV-2 infection:

- A longer ventilation period, leading to greater mechanical exposure to the risk of VAP [3].
- The frequency of comorbidities [3].
- The frequency of ARDS, which is associated with a higher incidence of VAP [6].
- A form of acquired immunosuppression related to SARS-CoV-2 infection,
- Organizational factors related to the fact that this is the first major pandemic in modern history.

The first studies published on this subject seem to confirm that COVID-19 patients are at greater risk of developing VAP [9–11]. Nevertheless, the sudden increase in workload in the centers participating in these studies may have had an impact on the incidence of VAP.

Although the Pays-de-la-Loire region was affected by this epidemic, it was not inundated. Our units had time to prepare, with beds, ventilators and nurses available, and in addition, we were able to accept patients from the areas that were most affected (the Grand-Est and Ile-de-France regions). This regional particularity decreases the amount of confounding factors related to the exceedance of ICU capacities [12].

Therefore, we wished to conduct a retrospective study to epidemiologically and microbiologically describe VAP that had occurred since the beginning of the epidemic in a population of patients with severe COVID-19 hospitalized in our geographical region.

## Methods

All patients diagnosed with COVID-19, who were admitted to all the ICUs in the Pays-de-la-Loire region between March 1, 2020, and May 18, 2020, were screened. The study involved seven general hospitals including two university teaching hospitals.

Patients were required to be over 18 years of age, have a positive RT-PCR for SARS-CoV-2 and have been receiving IMV for more than 48 h. Exclusion criteria were: pregnancy, guardianship and refusal to participate (by the patient or relatives).

After inclusion, data were retrospectively collected from an electronic Case Report Form (eCRF), filled out by the investigators in each study center. The CRF included demographic and clinical characteristics of the patients at ICU admission, data related to the disease course, ICU treatments, outcomes and, finally, data related to each VAP episode (date of onset, sampling method, implicated germ(s), related complication(s), etc.).

The diagnosis of VAP was established by the team in charge of the patient if hospital-acquired pneumonia was diagnosed after at least 48 h of mechanical ventilation, based on criteria adapted from the European Centre for Disease Prevention and Control (ECDC) recommendations [13]:

1. Radiological signs: two successive chest radiographs or chest CT scans showing new or progressive lung infiltrates
2. And at least one of the following systemic signs:
  Body temperature > 38.3 °C with no other cause
  Leukocytes < 4000/mm^3^ or > 12,000/mm^3^
    Body temperature > 38.3 °C with no other cause
3. And at least one of the following respiratory signs:
  New onset of purulent sputum or change in character of sputum (color, odor, quantity, consistency)
  Worsening gas exchange (e.g., O2 desaturation or increased oxygen requirements or increased ventilation demand)
4. And at least one of the following microbiological criteria:
  Positive quantitative culture from minimally contaminated LRT specimen (PN 1 type in ECDC classification [13]) using plugged telescopic catheter with a threshold of 10^3^ CFU/mL or a bronchoalveolar lavage with a threshold of 10^4^ CFU/ml.
  Positive quantitative culture from possibly contaminated LRT specimen (PN 2 type in ECDC classification [13]) using blind endotracheal aspirate with a threshold of 10^6^ CFU/ml.
  Positive growth in culture of pleural fluid (PN 3 type in ECDC classification [13])

Whenever the first three criteria were present, a quantitative microbiological sample was taken. Microbiological documentation was mandatory to establish VAP diagnosis.

The choice of sampling technique was different among the sites and depended on local procedures. Abscesses were established by CT scan, empyema by thoracentesis with pleural exudate and the presence of bacteria in the culture.

We reported means (± standard deviation) for quantitative variables and frequency, and percentages for qualitative variables.

The characteristics of patients who had at least one poly-microbial VAP were compared to those of patients with a mono-microbial VAP using the Wilcoxon tests for quantitative data and the Chi-squared test (or Fisher’s test) for categorical data.

Known respiratory diseases and immunocompromised status were grouped according to the following rules:

Known respiratory disease: asthma, chronic obstructive pulmonary disease (COPD), obstructive sleep apnea syndrome (OSAS), pulmonary emphysema or interstitial lung disease.

Immunocompromised status: hematologic malignancies, Human Immunodeficiency Virus infection, immunosuppressive therapy or long-term corticosteroid therapy.

The incidence density of VAP was calculated according to two different methods:

- *Type 1* VAP incidence density (up to the first VAP) was calculated as follows: (number of patients with at least one occurrence of VAP/total amount of days of invasive ventilation until the first VAP) * 1000.
- *Type 2* VAP incidence density (in episodes) was calculated as follows: (total number of VAP episodes/total amount of days of invasive ventilation) * 1000.

Factors associated with the occurrence of VAP were evaluated with the Fine–Gray method. Cumulative incidence of VAP was analyzed considering death and extubation as competing risk events. Only age, sex and obesity were included in multivariate analysis. Vasopressor use and Extra-Corporeal Membrane Oxygenation (ECMO) were not included because we did not know whether they were started before or after VAP diagnosis.

Patient status was censored as of May 20, 2020.

A less than 0.05 p-value was considered statistically significant.

All the analysis was done using R software (version 3.6.3).

This study was authorized by the local ethics committee and was declared to the French data protection authority (CNIL). Patients received an information note and were given the opportunity to object to data collecting.

## Results

This study included 194 patients placed on IMV for COVID-19. Data for six patients were not analyzed: three were under guardianship, two were admitted to the ICU after the May 18, 2020, deadline, and one withdrew his consent. We then analyzed the data for 188 patients whose characteristics at ICU admission are described in Table 1.

**Table 1:** Demographic and clinical characteristics of patients at ICU admission. Risk factors of developing one VAP (univariate analysis, Fine–Gray model considering death and extubation as competing risk events)

	All patients	No VAP	At least one VAP	sHR [95%CI], p-value
Headcount	188	96	92	
**Baseline and demographic**				
Mean age (± SD)—years	63.9 (± 11.4)	62.5 (± 12.9)	65.3 (± 9.5)	1.01 [0.99–1.03], * p* = 0.170
Male—no. (%)	147 (78.2)	67 (69.8)	80 (87)	**2.12 [1.14–3.96], p = 0.018**
Obesity—no./total no. (%)	69/182 (37.9)	40/92 (43.5)	29/90 (32.2)	0.67 [0.40–1.12], * p* = 0.130
Hypertension—no. (%)	90 (47.9)	47 (49)	43 (46.7)	0.95 [0.64–1.43], * p* = 0.820
Diabetes—no. (%)	50 (26.6)	25 (26)	25 (27.2)	0.96 [0.62–1.49], * p* = 0.860
Current smoker—no. (%)	10 (5.6)	7 (7.3)	3 (3.3)	-
Asthma—no. (%)	13 (6.9)	6 (6.2)	7 (7.6)	Known respiratory disease 1.39 [0.81, 2.41], * p* = 0.240
COPD—no. (%)	6 (3.2)	1 (1)	5 (5.4)	
OSAS—no. (%)	9 (4.8)	5 (5.2)	4 (4.3)	
Pulmonary emphysema—no. (%)	4 (2.1)	-	4 (4.3)	
Interstitial lung disease—no. (%)	3 (1.6)	1 (1)	2 (2.2)	
Cirrhosis—no. (%)	3 (1.6)	2 (2.1)	1 (1.1)	-
Chronic kidney failure—no. (%)	8 (4.3)	6 (6.2)	2 (2.2)	-
Chronic heart failure—no. (%)	13 (6.9)	8 (8.3)	5 (5.4)	-
Cancer—no. (%)	11 (5.9)	5 (5.2)	6 (6.5)	-
Hematologic malignancies—no. (%)	5 (2.7)	4 (4.2)	1 (1.1)	Immunocompromised status 0.68 [0.32, 1.43], * p* = 0.310
HIV infection—no. (%)	4 (2.1)	2 (2.1)	2 (2.2)	
Immunosuppressive therapy—no. (%)	7 (3.7)	3 (3.1)	4 (4.3)	
Long-term corticosteroid therapy—no. (%)	3 (1.6)	2 (2.1)	1 (1.1)	
**On arrival in the ICU**				
Mean SAPS II (± SD)	41 (± 13.5)	41.1 (± 13.8)	40.9 (± 13.3)	1.00 [0.98–1.01], * p* = 0.700
Mean SOFA (± SD)	5.8 (± 2.7)	5.8 (± 2.7)	5.7 (± 2.7)	1.00 [0.92–1.09], * p* = 0.960
Mean PaO2/FiO2 (± SD)—mmHg	150 (± 63.4)	150 (± 63.6)	150 (± 63.6)	1.00 [1.00–1.00], *p* = 0.850

ICU, intensive care unit; VAP, ventilator-associated pneumonia; sHR, sub-hazards ratio; CI, confidence interval; SD, standard deviation; COPD, chronic obstructive pulmonary disease; OSAS, obstructive sleep apnea syndrome; HIV, Human Immunodeficiency Virus; SAPS II, Simplified Acute Physiology Score II; SOFA, sepsis-related organ failure assessment; PaO2, arterial partial pressure of oxygen; FiO2, fraction of inspired oxygen

The average age was 63.9 years (± 11.4), with a clear predominance of males (78.2%). A large majority (76.4%) was overweight (body mass index (BMI) > 25), and 37.9% were obese (BMI > 30). Nearly half (47.9%) had hypertension, and a quarter (26.6%) suffered from diabetes. A total of 17% reported at least one known respiratory disease. In addition, 8% were immunocompromised (malignant hemopathy, immunosuppressive therapy, long-term corticosteroid therapy, Human Immunodeficiency Virus infection) and 5.9% were treated for cancer. A total of 75.5% presented one or more comorbidities. At ICU admission, the mean Simplified Acute Physiology Score II (SAPS2) score was 41 (± 13.5), and the mean Sepsis-related Organ Failure Assessment (SOFA) score was 5.8 (± 2.7). The average PaO_2_/FiO_2_ ratio was 150 (± 63.4) mmHg indicating severe hypoxemia. The characteristics related to COVID-19 are described in Table 2.

**Table 2:** COVID-19: disease course, in-hospital treatments and outcome. Risk factors of developing one VAP (univariate analysis, Fine–Gray model considering death and extubation as competing risk events)

	All patients	No VAP	At least one VAP	sHR [95%CI], *p* value
Headcount	188	96	92	
**Disease course and in-hospital treatments**				
Mean period from symptom onset to hospital admission (± SD)—days	6.4 (± 8.0)	6.4 (± 6.2)	6.4 (± 9.6)	-
Mean period from symptom onset to COVID-19 diagnosis (± SD)—days	7.7 (± 4.2)	7.6 (± 4.3)	7.7 (± 4)	-
Transfer from inundated area—no. (%)	47 (25.0)	20 (20.8)	27 (29.3)	-
Proven co-infection—no. (%)	21 (11.2)	11 (11.5)	10 (10.9)	-
Initial empirical antibiotic therapy—no. (%)	169 (89.9)	87 (90.6)	82 (89.1)	0.71 [0.34–1.48], *p* = 0.360
Antiviral therapy—no. (%)	112 (59.6)	57 (59.4)	55 (59.8)	0.95 [0.63–1.43], *p* = 0.790
Lopinavir/ritonavir—no. (%)	64 (34.0)	31 (32.3)	33 (35.9)	1.04 [0.69–1.56], *p* = 0.860
Remdesivir—no. (%)	10 (5.3)	5 (5.2)	5 (5.4)	1.00 [0.43–2.33], *p* = 1.000
Hydroxychloroquine—no. (%)	42 (22.3)	22 (22.9)	20 (21.7)	0.94 [0.58–1.52], *p* = 0.800
Immunomodulatory drugs—no. (%)	22 (11.7)	10 (10.4)	12 (13)	
Corticosteroids—no. (%)	21 (11.2)	10 (10.4)	11 (12)	1.00 [0.57–1.76], *p* = 1.000
Tocilizumab—no. (%)	1 (0.5)	-	1 (1.1)	–
Mean period between hospital admission and intubation (± SD)—days	2.6 (± 7.5)	2.5 (± 5.3)	2.7 (± 9.2)	–
Vasopressor support—no. (%)	128 (68.1)	57 (59.4)	71 (77.2)	**1.94 [1.22–3.09], p = 0.005**
Average duration of mechanical ventilation (± SD)—days	22.2 (± 16.7)	14.5 (± 11.3)	30.3 (± 17.7)	**-**
ECMO—no. (%)	18 (9.6)	4 (4.2)	14 (15.2)	**3.09 [1.59, 6.03], p = 0.001**
**Outcome**				
Deceased on May 20th—no. (%)	54 (28.7)	26 (27)	28 (30.4)	
Discharged alive from ICU on May 20th—no. (%)	115 (61.2)	64 (66.7)	51 (55.4)	
Still in ICU on May 20th—no. (%)	19 (10.1)	6 (6.3)	13 (14.1)	

A significant proportion of our patients (25%) received initial treatment in overcrowded centers (located in heavily affected areas) before being transferred to our hospitals.

Mean time between symptom onset and hospital admission was 6.4 (± 8.0) days, and the mean time between hospital admission and intubation was 2.6 (± 7.5) days.

One hundred and sixty-nine patients (89.9%) received an initial empirical antibiotic therapy for pneumonia. The most frequently used molecules were third-generation cephalosporins (3GC) (82.9%), spiramycin (67.5%), amoxicillin-clavulanate (12.4%), azithromycin (6.5%) and piperacillin-tazobactam (5.3%).

Twenty-one (11.2%) patients had proven coinfections which are listed in Table 3.

**Table 3:** List of co-infection-related isolates

Total number of isolates	25
Staphylococcus aureus—no. (%)	6 (24.0)
Enterobacteria—no. (%)	5 (20.0)
Streptococcus pneumoniae—no. (%)	3 (12.0)
Haemophilus influenzae—no. (%)	3 (12.0)
Other viruses—no. (%)	3 (12.0)
Other gram-negative bacteria—no. (%)	3 (12.0)
Other gram-positive bacteria—no. (%)	2 (8.0)

They were mainly due to *Staphylococcus aureus*, enterobacteria, *Streptococcus pneumoniae* and *Haemophilus influenzae*. Only one *Pseudomonas aeruginosa* was identified and three viruses (two rhinoviruses and one bocavirus).

One hundred and twelve patients (59.6%) were treated with at least one antiviral drug:

sixty-four (34%) received a lopinavir/ritonavir combination, forty-two (22.3%) received hydroxychloroquine, and ten (5.3%) received remdesivir. Twenty-one (11.2%) were treated with corticosteroid therapy.

One hundred and twenty-eight (68.1%) received vasopressor therapy, and eighteen (9.3%) were treated with Venovenous Extra-Corporeal Membrane Oxygenation (VV-ECMO).

The total mean duration of intubation for all patients (whether or not they were still in the ICU) was 22.2 (± 16.7) days, which is a total of 4181 days of ventilation. A total of 343 microbiological samples were taken in order to confirm a suspected VAP (i.e., on average, one every 12.2 days of IMV), and we identified 141 microbiologically documented VAP episodes.

We identified at least one microbiologically documented VAP in 92 (48.9%) patients. Median time between intubation and the onset of the first VAP was 10 days.

The *type 1* incidence density was 39.0 VAP per 1000 days of IMV. Thirty-eight patients (20% of all) experienced multiple VAP episodes, twenty-six of whom had 2, eleven had 3, and one had 4 documented VAP episodes. Therefore, when all VAP episodes are considered, the *type 2* incidence density was 33.7 VAP per 1000 days of IMV.

Sampling techniques used to confirm pneumonia were blind endotracheal aspiration in sixty cases (42.6%), bronchoalveolar lavage (BAL) in fifty cases (35.4%), a plugged telescopic catheter in thirty cases (21.3%) and a pleural effusion analysis in one case (0.7%) (the patient had pneumonia with a bronchopleural fistula on CT scan and concomitant pleural effusion). Multi-microbial VAP accounted for 39.0% of all VAP. We identified a total of 205 pathogens listed in Table 4.

**Table 4:** List of ventilator-associated pneumonia-related isolates

VAP group	All	Early (< 5 days *)	Late (≥ 5 days *)	Mono-microbial	Poly-microbial
**Headcount**	143	14	129	86	55
**Total number of isolates**	205	17	188	86	119
Enterobacteria—no. (%)	102 (49.8)	6 (35.3)	96 (51.1)	38 (44.2)	64 (53.8)
Escherichia coli—no. (%)	26 (12.7)	3 (17.6)	23 (12.2)	11 (12.8)	15 (12.6)
Klebsiella pneumoniae—no. (%)	16 (7.8)	–	16 (8.5)	8 (9.3)	8 (6.7)
Serratia marcescens—no. (%)	12 (5.9)	–	12 (6.4)	6 (7.0)	6 (5.0)
Enterobacter cloacae—no. (%)	10 (4.9)	–	10 (5.3)	3 (3.5)	7 (5.9)
Citrobacter koseri—no. (%)	9 (4.4)	1 (5.9)	8 (4.3)	3 (3.5)	6 (5.0)
Hafnia alvei—no. (%)	8 (3.9)	1 (5.9)	7 (3.7)	4 (4.7)	4 (3.4)
Klebsiella aerogenes—no. (%)	8 (3.9)	–	8 (4.3)	3 (3.5)	5 (4.2)
Klebsiella oxytoca—no. (%)	5 (2.4)	–	5 (2.7)	–	5 (4.2)
Proteus mirabilis—no. (%)	4 (2.0)	–	4 (2.1)	–	4 (3.4)
Other Proteus—no. (%)	2 (1.0)	1 (5.9)	1 (0.5)	–	2 (1.7)
Citrobacter freundii—no. (%)	1 (0.5)	–	1 (0.5)	–	1 (0.8)
Other Enterobacter—no. (%)	1 (0.5)	–	1 (0.5)	–	1 (0.8)
Pseudomonas aeruginosa—no. (%)	31 (15.1)	1 (5.9)	30 (16.0)	19 (22.1)	12 (10.1)
Staphylococcus aureus—no. (%)	28 (13.7)	4 (23.5)	24 (12.8)	15 (17.4)	13 (10.9)
Enterococcus faecalis—no. (%)	11 (5.4)	–	11 (5.9)	3 (3.5)	8 (6.7)
Other Gram-negative bacteria—no. (%)	21 (10.2)	2 (11.8)	19 (10.1)	8 (9.3)	13 (10.9)
Stenotrophomonas maltophilia—no. (%)	8 (3.9)	–	8 (4.3)	4 (4.7)	4 (3.4)
Haemophilus—no. (%)	5 (2.4)	1 (5.9)	4 (2.1)	–	5 (4.2)
Acinetobacter baumanii—no. (%)	4 (2.0)	–	4 (2.1)	2 (2.3)	2 (1.7)
Other Pseudomonas and related—no. (%)	2 (1.0)	–	2 (1.1)	2 (2.3)	–
Other non-Enterobacteria—no. (%)	2 (1.0)	1 (5.9)	2 (1.1)	1 (1.1)	1 (0.8)
Obligate anaerobe—no. (%)	1 (0.5)	–	1 (0.5)	–	1 (0.8)
Other Gram-positive bacteria—no. (%)	12 (5.9)	4 (23.5)	8 (4.3)	3 (3.4)	9 (7.5)
Other Streptococci—no. (%)	5 (2.4)	2 (11.8)	3 (1.6)	1 (1.2)	4 (3.4)
Streptococcus agalactiae—no. (%)	2 (1.0)	1 (5.9)	1 (0.5)	–	2 (1.7)
Streptococcus pneumonia—no. (%)	2 (1.0)	1 (5.9)	1 (0.5)	1 (1.2)	1 (0.8)
Enterococcus faecium—no. (%)	1 (0.5)	–	1 (0.5)	–	1 (0.8)
Corynebacterium—no. (%)	1 (0.5)	–	1 (0.5)	–	1 (0.8)

* Days of invasive mechanical ventilation

VAP, ventilator-associated pneumonia

Enterobacteria represented 49.8% of the isolates, *Pseudomonas aeruginosa* accounted for 15.1%, *Staphylococcus aureus* for 13.7%, followed by other gram-negative bacteria (*Stenotrophomonas maltophilia*, *Haemophilus*, *Acinetobacter baumannii*, other *Pseudomonas*, etc.) 10.2%, other gram-positive bacteria (*Streptococcus pneumoniae, Streptococcus agalactiae*, corynebacteria, *Enterococcus faecium*, etc.) 5.9%, and *Enterococcus faecalis* 5.4%.

Note: *Aspergillus fumigatus* was present in culture in 5 cases and treatment was initiated in all cases.

The ecological status was different depending on the time of occurrence, with a higher proportion of gram-positive bacteria in early VAP (47.0% versus 22.8% in late VAP) and a clearer predominance of gram-negative bacteria in late VAP (77.2%).

Pseudomonas aeruginosa was less often involved in poly-microbial VAP (10.1% of cases) than in mono-microbial VAP (22.1% of cases).

Characteristics of patients with poly-microbial VAP are described in Table 5. Patients with poly-microbial VAP were older (68.5 vs. 62.8 years, *p* = 0.005) than those with exclusively mono-microbial VAP, fewer of them had received prior empirical antibiotic therapy at the initial phase (77.5% versus 98.1%, *p* = 0.002), and they were on IMV for a longer period (37.8 ± 20.6 vs. 24.6 ± 12.5 days) and were therefore more likely to be still hospitalized in intensive care at the end of the study period (25.0% vs 5.8%, *p* = 0.009).

**Table 5:** Characteristics of patients at ICU admission, COVID-19 disease course, in-hospital treatments and outcome: comparisons by VAP type (mono-microbial vs. poly-microbial)

	Only mono-microbial VAP	At least one poly-microbial VAP	*p* value
Headcount	52	40	
**Baseline and demographic**			
Mean age (± SD)—years	62.8 (± 10.1)	68.5 (± 7.5)	**0.005**
Male—no. (%)	44 (84.6)	36 (90.0)	0.447
Obesity—no./total no. (%)	19/50 (37.3)	10/39 (25.6)	0.273
Hypertension—no. (%)	21 (40.4)	22 (55.0)	0.164
Diabetes—no. (%)	15 (28.8)	10 (25.0)	0.681
Current smoker—no. (%)	1 (1.9)	2 (5.0)	0.578
Asthma—no. (%)	5 (9.6)	2 (5.0)	0.695
COPD—no. (%)	3 (5.8)	2 (5.0)	1.000
OSAS—no. (%)	2 (3.8)	2 (5.0)	1.000
Pulmonary emphysema—no. (%)	1 (1.9)	3 (7.5)	0.313
Interstitial lung disease—no. (%)	–	2 (5.0)	0.186
Cirrhosis—no. (%)	1 (1.9)	–	1.000
Chronic kidney failure—no. (%)	2 (3.8)	–	0.503
Chronic heart failure—no. (%)	3 (5.8)	2 (5.0)	1.000
Cancer—no. (%)	2 (3.8)	4 (10.0)	0.398
Hematologic malignancies—no. (%)	-	1 (2.5)	0.435
HIV infection—no. (%)	1 (1.9)	1 (2.5)	1.000
Immunosuppressive therapy—no. (%)	4 (7.7)	–	0.130
Long-term corticosteroid therapy—no. (%)	1 (1.9)	–	1.000
**On arrival in the ICU**			
Mean SAPS II (± SD)	38.9 (± 12.7)	44.1 (± 13.8)	0.098
Mean SOFA (± SD)	5.6 (± 2.7)	6.0 (± 2.7)	0.553
Mean PaO2/FiO2 (± SD)—mmHg	143.8 (± 59.2)	158.7 (± 69.3)	0.282
**Disease course and in-hospital treatments**			
Mean period from symptom onset to hospital admission (± SD)—days	5.8 (± 12.5)	7.1 (± 3.3)	0.827
Mean period from symptom onset to COVID-19 diagnosis (± SD)—days	7.4 (± 3.8)	8.0 (± 4.3)	0.398
Transfer from inundated area—no. (%)	15 (28.8)	12 (30.0)	0.904
Proven co-infection—no. (%)	4 (7.7)	6 (15.0)	0.264
Initial empirical antibiotic therapy—no. (%)	51 (98.1)	31 (77.5)	**0.002**
Antiviral therapy—no. (%)	34 (65.4)	21 (52.5)	0.212
Lopinavir/ritonavir—no. (%)	21 (40.4)	12 (30.0)	0.303
Remdesivir—no. (%)	4 (7.7)	1 (2.5)	0.383
Hydroxychloroquine—no. (%)	9 (17.3)	11 (27.5)	0.240
Immunomodulatory drugs—no. (%)	7 (14)	5 (11.9)	-
Corticosteroids—no. (%)	6 (11.5)	5 (12.5)	0.888
Tocilizumab—no. (%)	1 (1.9)	–	–
Mean period between hospital admission and intubation (± SD)—days	3.2 (± 12.0)	2.1 (± 2.8)	0.381
Vasopressor support—no. (%)	37 (71.2)	34 (85.0)	0.117
Average duration of mechanical ventilation (± SD)—days	24.6 (± 12.5)	37.8 (± 20.6)	**0.001**
ECMO—no. (%)	7 (13.5)	7 (17.5)	0.593
**Outcome**			
Deceased on May 20th—no. (%)	16 (30.8)	12 (30.0)	0.937
Discharged alive from ICU on May 20th—no. (%)	33 (63.5)	18 (45.0)	–
Still in ICU on May 20th—no. (%)	3 (5.8)	10 (25.0)	**0.009**

VAP was associated with bacteremia in 15 (10.6%) cases (only bacteremia with the same germ as the one responsible for the VAP is reported here). Pneumonia was complicated by an abscess in two cases (1.4%) and by thoracic empyema in five cases (3.5%).

**Fig. 1:**
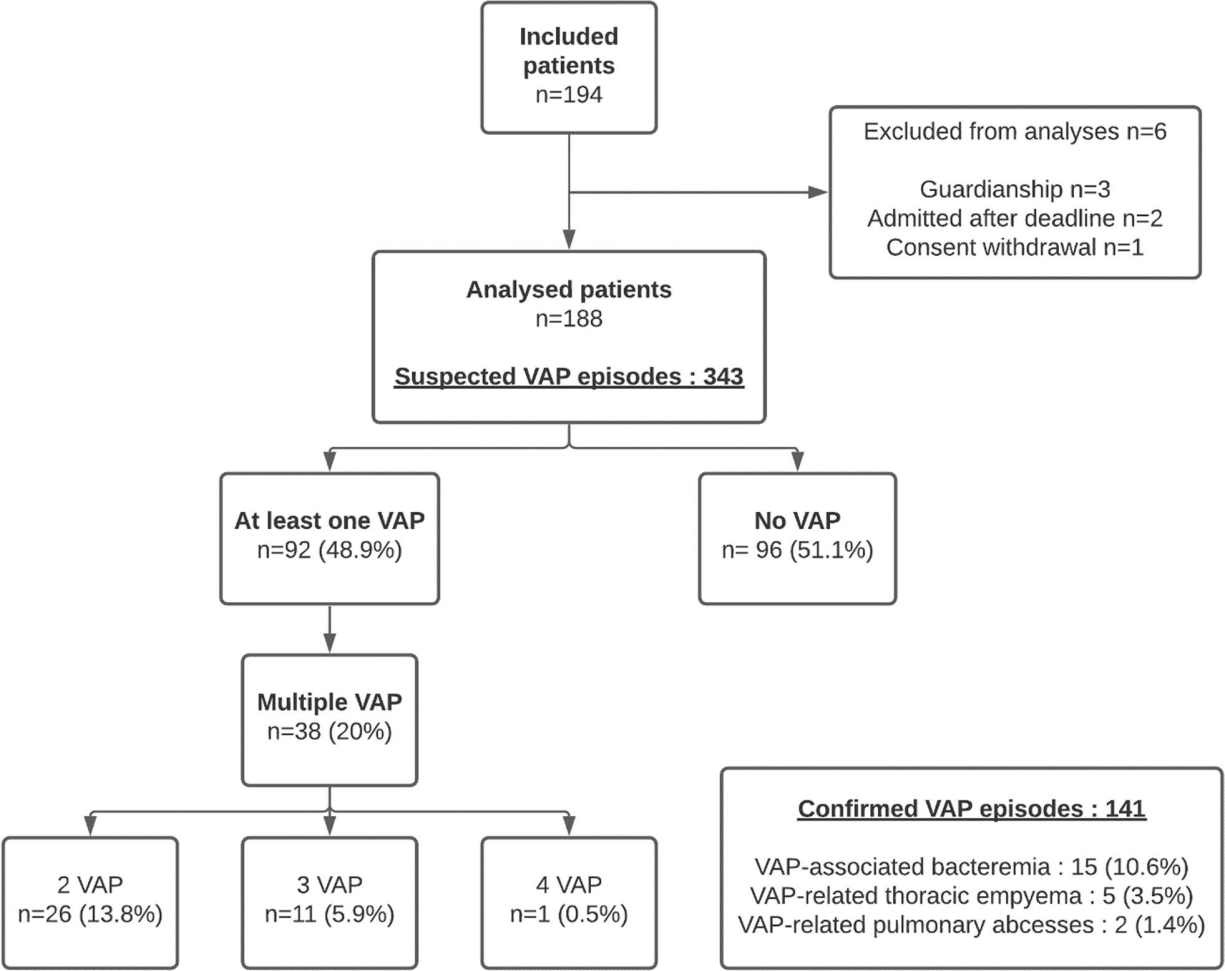
Number of patients and VAP count

For each situation, the number of patients is indicated in Fig. 1.

Regarding the antibiotic resistance profile, we identified only three methicillin-resistant *Staphylococcus aureus*.

Of the 92 patients who had one or more VAP, at least one enterobacterium was involved in 59 (64.1%) cases. We identified at least one 3GC-resistant enterobacterium in 31 (52.5%) of these 59 patients, and they had received prior 3GC therapy in 87.1% of the cases. Of the 28 others (no enterobacteria with a 3GC resistance mechanism identified), 64% had received prior 3GC therapy.

As of May 20, 2020, nineteen (10.1%) patients were still hospitalized in intensive care, one hundred and fifteen (61.2%) had been discharged from the ICU, and fifty-four (28.7%) had died in the ICU.

Four of the five patients with *Aspergillus fumigatus* in culture had died despite antifungal treatment, as well as the two patients who had a pulmonary abscess (the duration of antibiotic treatment set by clinicians was a minimum of 4 weeks in both cases), two of the four who had purulent pleurisy (one patient developed two episodes and died) and three of the fourteen who had a VAP-associated bacteremia episode (one patient presented two).

**Fig. 2:**
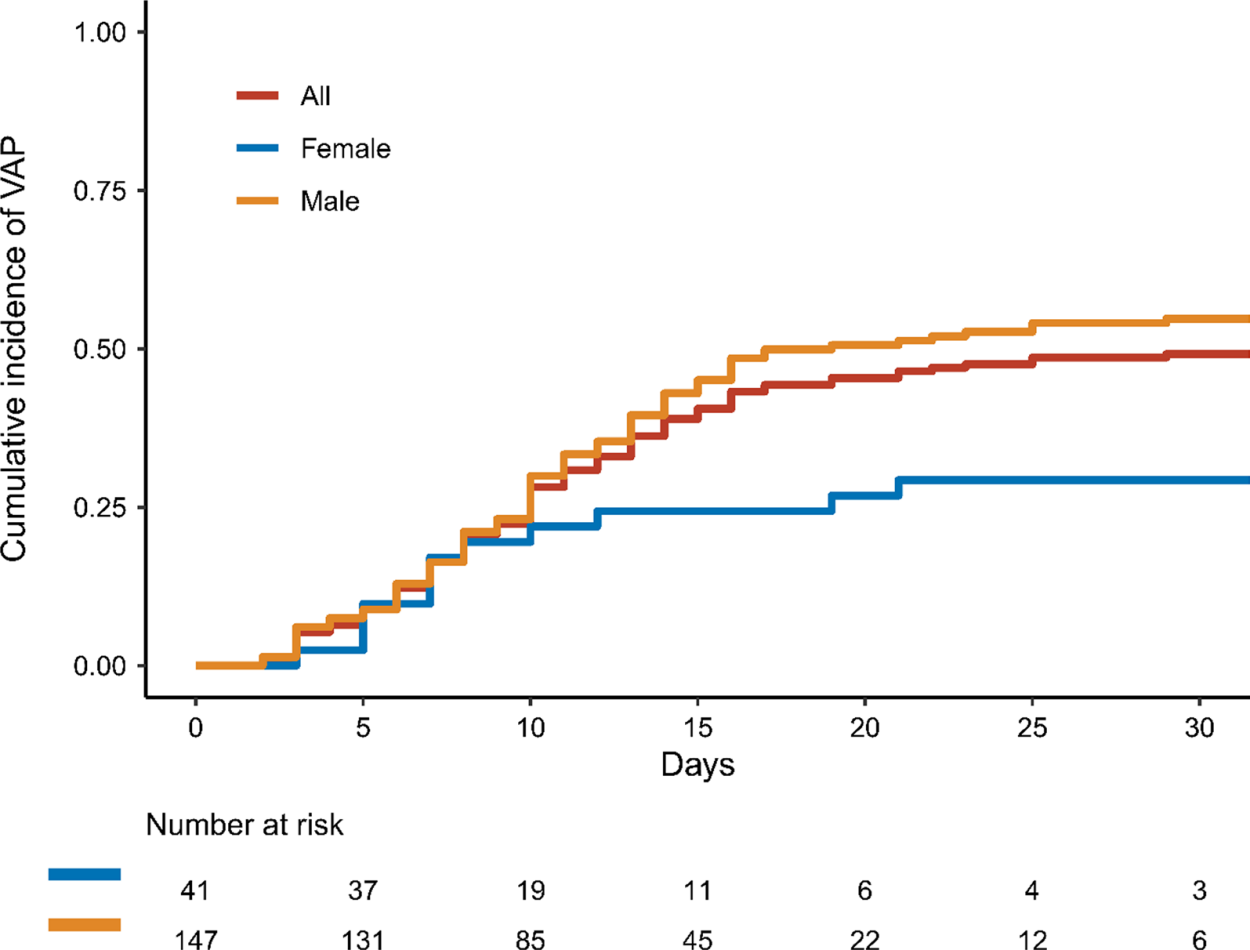
Cumulative incidence of VAP according to the patient's sex

In univariate Fine–Gray regression analysis (Tables 1 and 2), male sex (sHR 2.13 CI95% [1.14; 3.97] *p* = 0.018), use of ECMO (sHR 3.06 CI95% [1.58; 5.92] *p* = 0.001) and use of a vasopressor (sHR 1.95 [1.23. 3.10] *p* = 0.005) were associated with a significantly higher occurrence of VAP. There was no statistically significant relationship between VAP and age, obesity, hypertension, diabetes, known respiratory disease, immunocompromised status, SAPS2, SOFA score, initial PaO2/FiO2 ratio, initial empirical antibiotic therapy, antiviral therapy or corticosteroids. In the step-by-step multivariate analysis (Table 6), males were associated with a higher risk of developing VAP (sHR 2.25 CI95% [1.19–4.25] *p* = 0.013). The cumulative incidence of VAP according to the patient's sex is shown in Fig. 2.

**Table 6:** Multivariate analysis of risk factors for developing at least one VAP. Fine–Gray model considering death and extubation as competing risk events

Characteristic (complete model)	sHR	95%CI	*p* value
Male	**2.07**	**1.08–4.00**	**0.029**
Age	1.01	1.00–1.03	0.094
Obesity	0.78	0.47–1.31	0.360

Characteristic (step-by-step model)	sHR	95%CI	*p* value
Male	**2.24**	**1.18–4.26**	**0.013**
Age	1.02	1.00–1.03	0.082

## Discussion

### VAP incidence

Our data show a remarkably high incidence of VAP (crude incidence rate of 48.9% with an incidence density of 39.2 VAP per 1000 days of IMV). This is more than twice as high as was reported in a cohort of 2436 patients admitted to European intensive care units [4]. The incidence of VAP remains unusually high in our study compared to studies including only ADRS patients, which were ancillary studies to ACURASYS (28.9%) and PROSEVA (between 11.8 and 15.4 VAP per 1000 days of IMV) trials [6, 14]. In terms of recently published studies on COVID-19 patients, our results are quite similar to those reported by Maes et al. in a monocentric cohort of 81 mechanically ventilated patients (crude incidence of 48%) [10]. Our results are also relatively consistent with those of Rouzé et al. in a multicentric cohort of 568 patients [9]. They noted a crude incidence of 50.5% of Ventilator-Associated Lower Respiratory Tract Infection (VA-LRTI), defined using the same criteria as VAP but without any radiological signs. In these two retrospective studies, COVID-19 patients were about twice as likely to develop VAP compared to the control populations. Finally, in a monocentric retrospective study, Razazi et al. reported a crude incidence of 64% in a cohort of 90 COVID-19 patients [11].

Our cohort was quite similar to those of other studies on VAP in COVID-19 patients [9–11] except for sex ratio (21.8% females in our cohort and 18% for Razazi et al. vs. 31% for Maes et al. and 41.4% for Rouzé et al.), and it was not very different from other ARDS studies such as PROSEVA [15] and ACURASYS [5]. Our patients were slightly older (63.9 vs. 59 and 58 for PROSEVA [15] and ACURASYS [5], respectively), and the proportion of previously immunocompromised patients was slightly smaller (8.0% vs. 15.0% and 18% for PROSEVA [15] and ACURASYS [5], respectively).

As in other studies on VAP in COVID-19 patients [9–11], immunocompromised patients represented a modest proportion of our severe COVID-19 population. Moreover, immunodeficiency was not associated with the occurrence of VAP in our study.

Several factors may have contributed to this high incidence of VAP observed in our study: (a) The duration of mechanical ventilation was prolonged in our cohort compared to classical ARDS patients (22 days vs 18) [15], but it could be as much a cause as a consequence, (b) we included patients with VV-ECMO (9.6%) who are known to be at higher risk of VAP [17], but although they experienced more VAP, the type 1 incidence density remained very high when the analysis was repeated without these patients (35.5 instead of 39.0/1000 days of mechanical ventilation), (c) we included patients initially transferred from heavily affected areas, but we found no difference in the incidence of VAP in this population, (d) few patients were treated with corticosteroids (only 11.2% of our patients), and it was not associated with an increased risk of VAP.

Nevertheless, we must consider the influence of SARS-CoV-2 on immunity. Studies have already shown that COVID-19 infection leads to a decrease in lymphocytes which affects the different lymphocyte subtypes: CD4 and CD8 [18], natural killer (NK), regulatory and cytotoxic T lymphocytes [19] and B lymphocytes [20]. This hypothesis is primarily supported by the unusual frequency of VAP complications (bacteremia, abscesses and empyema). In previous published studies [9–11], abscesses, empyema and bacteremia were not reported.

Furthermore, diagnosing VAP is challenging and even more so in COVID-19 patients [21], notably due to: (a) difficulties interpreting chest X-rays in these patients who present with bilateral infiltrates from admission to the ICU; (b) a greater frequency of chest CT scans; (c) frequent bacterial colonization; and (d) repeated variations in respiratory parameters. These reasons could have led to an overestimation of VAP incidence, but we only reported microbiologically documented VAP (PN 1, PN 2 and PN 3 types in the ECDC classification [13]).

### Microbial ecology

We also highlighted a particular microbial ecology, since enterobacteria were responsible for 44.2% of the cases of mono-microbial pneumonia and represented 49.8% of all the isolated pathogens, followed by other gram-negative bacilli which accounted for 25.3% (*Pseudomonas aeruginosa*: 15.1%) and gram-positive cocci which accounted for 24.9% (13.7% *Staphylococcus aureus*). As expected, non-fermenting bacilli (i.e., *Pseudomonas aeruginosa, Acinetobacter baumanii, Stenotrophomonas maltophilia and other Pseudomonas* species) were almost absent in early VAP (< 5 days), while they were more frequent (23.4%) in late VAP (> 5 days). Interestingly, microbiological isolates from mono-microbial and poly-microbial VAP were not very different with the exception of a lower frequency of *Pseudomonas aeruginosa* in poly-microbial VAP. However, we found a higher rate of poly-microbial infection (39.0%) than other studies on VAP in COVID-19 patients (22% by Razazi et al [11] and 9.8% by Rouzé et al [9]). In their study on ARDS patients in 2008, Forel et al. found that the most common bacteria were non-fermenting gram-negative bacilli at 40%, followed by enterobacteria (29%) and *Staphylococcus aureus* (21%) [6]. In the PROSEVA cohort [14], the authors reported enterobacteria in 33.9% of their isolates. This difference may reflect a peculiarity of COVID-19 as a basic trend in the changes in the ecology of VAP.

Data on the microbiological ecology of VAP in COVID-19 patients are conflicting. In the Rouzé et al. study, they found no difference between COVID-19 patients, influenza patients and patients with no viral infection [9] (50.5%, 50.4% and 48.9% of enterobacteria, respectively) which seems to be consistent with the hypothesis of a basic trend in the changes in VAP ecology. On the contrary, Razazi et al. reported a higher incidence of enterobacteria [11]. In their study, Maes et al. concluded that there was no significant difference in VAP microbiological ecology between COVID-19 patients and non-COVID-19 patients, but an analysis of their figures shows a higher proportion of *Escherichia coli*, *Serratia* and *Klebsiella*.

### VAP risk factors analysis

This is the first multicentric study analyzing risk factors for VAP occurrence in the specific population of COVID-19 patients. Razazi et al. identified risk factors globally in a cohort including COVID-19 ARDS patients and non-COVID-19 ARDS patients [11].

First, initial empirical antibiotic therapy largely prescribed in our study was not associated with a higher risk of VAP. On the contrary, the lack of initial anti-infective treatment seemed to be associated with an increased risk of poly-microbial VAP. However, with the need for routine introduction of empirical antibiotic therapy in patients suffering from SARS-CoV-2 pneumonia, the rate of coinfections should be weighed against the risk of selecting resistant bacteria in these patients who have an extended length of stay and are likely to suffer from one or more episodes of VAP. In our study, at least one 3GC-resistant enterobacterium was detected in 52.5% of the patients who had at least one enterobacterium-VAP. Procalcitonin was not routinely measured in the ICUs that participated in our study, although it could be a helpful tool in the decision regarding early use of antibiotics. However, procalcitonin levels are generally elevated in COVID-19 ICU inpatients [22], and data are needed to assess a possible specific threshold.

Secondly, we found a significant association between male sex and the occurrence of at least one VAP in both univariate and multivariate analysis, taking into account death and extubation as competitive events. It is now well documented that men are more likely to develop severe forms of COVID-19 and are vastly over-represented in the ICU [23]. Differences in immunological response related to genetics or hormonal status could be one explanation [24]. In cohorts with large numbers of patients, Dananche et al. (134 510 patients) [25] and Rello et al. (9080 patients) [26] already identified sex as a risk factor of developing VAP.

Interestingly, Razazi et al. also reported that male sex was a risk factor for VAP after adjusting for death and extubation [11]. They analyzed their entire cohort including COVID-19 ARDS patients and non-COVID-19 ARDS patients. Whether or not this risk factor was more significant in COVID-19 ARDS patients than in non-COVID-19 ARDS patients was not specified.

Identifying sex as a VAP-associated factor in our cohort of 188 patients may be an indication of a sex-related immunologic difference exacerbated by SARS-CoV-2 infection.

Moreover, considering Fig. 2 and the fact that sex-specific curves diverge only as of the 10th day of ventilation, we can hypothesize that COVID-19-related immune perturbations could last longer in males than in females. In our opinion, these hypotheses are worth exploring.

Despite the fact that it is a well-established risk factor for developing VAP, we did not include the duration of mechanical ventilation in the multivariate analysis. We made this choice because we wanted to identify other potential risk factors, and a higher duration of mechanical ventilation might also have been a cause or a consequence of VAP, which would have resulted in an artificially strengthened association that might have overwhelmed others.

We admit that our study has some biases. This is a retrospective study conducted without strict harmonization of treatments or VAP prophylaxis bundles. However, daily exchanges between intensive care physicians of our region allowed us to ensure relative homogeneity in the global care of COVID-19 patients. Since an independent committee did not adjudicate the diagnosis of VAP, we may have overestimated the incidence, but to limit this bias, we included only episodes microbiologically established by at least semi-quantitative sampling or positive growth in pleural fluid culture in one case. In addition, although all the patients had a positive RT-PCR test, we cannot affirm that 100% had established pneumonia due to SARS-CoV-2 since some of them may have been receiving IMV for another reason (cardiogenic shock, severe pancreatitis, etc.). Chest CT scan was not performed in all our patients, resulting in a lack of information regarding the initial pulmonary parenchymal involvement.

The main strengths of our study are its multicentric nature and a well-sized cohort of patients during the first wave of the epidemic that was highly geographically inclusive in a region where we had time to adapt the size of the paramedical staff to the surplus of patients (with a ratio below 2 patients for 1 nurse) and where we were relatively spared. A sudden influx of patients quickly puts a strain on the healthcare system as individual workload increases resulting in an augmented risk of complications [27], and this could have impacted other studies.

## Conclusions

Our study shows an unusually high incidence of VAP in patients mechanically ventilated for severe COVID-19 in a non-inundated area. As previously published studies, a higher proportion of enterobacteria were found than in non-COVID-19 ARDS cohorts.

Pneumonia-related bacteremia and complications (abscess, empyema) are not uncommon. Therefore, in the absence of improvement, the clinician should probably focus on ruling out these complications.

Finally, our conclusions remain to be confirmed, especially since management practices for COVID-19 patients in the ICU are constantly evolving: The risk of infection needs to be addressed with the increasing use of non-invasive oxygen delivery techniques and the use of corticosteroids in severe COVID-19 patients [28].

## Data Availability

The datasets used and/or analyzed during the current study are available from the corresponding author on reasonable request.

## Abbreviations

ICU: Intensive care unit
IMV: Invasive mechanical ventilation
VAP: Ventilator-associated pneumonia
RT-PCR: Reverse transcriptase polymerase chain reaction
eCRF: electronic Case Report Form
ECDC: European Centre for Disease Prevention and Control (ECDC)
HR: Hazard ratio
ARDS: Acute respiratory distress syndrome
CNIL: “Commission Nationale de l’informatique et des libertés”: French data protection authority
BMI: Body mass index
SAPS2: Simplified Acute Physiology Score II
SOFA: Sepsis-related organ failure assessment
3GC: Third-generation cephalosporins
VV-ECMO: Venovenous Extra-Corporeal Membrane Oxygenation
BAL: Bronchoalveolar lavage
NK: Natural killer lymphocyte sub-type
VA-LRTI: Ventilator-Associated Lower Respiratory Tract Infection
